# A Simple, Robust and Efficient Computational Method for n-Octanol/Water Partition Coefficients of Substituted Aromatic Drugs

**DOI:** 10.1038/s41598-017-05964-z

**Published:** 2017-07-18

**Authors:** Asrin Bahmani, Saadi Saaidpour, Amin Rostami

**Affiliations:** 1 Department of Chemistry, Faculty of science, University of Kurdistan, Sanandaj, Iran; 2 Department of chemistry, Faculty of science, Islamic Azad University, Sanandaj Branch, Sanandaj, Iran

## Abstract

In this paper, multiple linear regression (MLR) was used to build quantitative structure property relationship (QSPR) of n-octanol-water partition coefficient (logP_o/w_) of 195 substituted aromatic drugs. The molecular descriptors were calculated for each compound by the VLifeMDS. By applying genetic algorithm/multiple linear regressions (GA/MLR) the most relevant descriptors were selected to build a QSPR model. The robustness of the model was characterized by the statistical validation and applicability domain (AD). The prediction results from MLR are in good agreement with the experimental values. The R^2^ and Q^2^_LOO_ for MLR are 0.9433, 0.9341. The AD of the model was analyzed based on the Williams plot. The effects of different selected descriptors are described.

## Introduction

Lipophilicity is the tendency of a compound to partition into a non-polar organic phase versus an aqueous phase. The typical quantitative descriptor of lipophilicity is the partition coefficient P of a given compound between two immiscible solvents^1^. Traditionally, n-octanol has been widely used as the non-polar phase and water as the polar phase. The partitioning value that is measured is termed logP_o/w_^2^.

1 $$log{{\rm{P}}}_{{\rm{o}}/{\rm{w}}}=log\frac{{C}_{o}}{{C}_{w}}\,$$

The n-octanol is considered a good mimic of phospholipids membrane characteristics because its nature is amphiphilic^3^. Among other physicochemical properties, lipophilicity plays a key role for molecular discovery activities in a variety of domains including, agrochemicals, cosmetics, material sciences, environmental chemistry, food chemistry, and particularly medicinal chemistry^4^. A correct estimation of logP_o/w_ is essential for the discovery and development of efficient therapeutic molecules^5^. Whereas lipophilicity cannot characterize the whole physicochemical nature of a compound, properties governing lipophilicity have a basic effect on the actions of organic molecules, such as drugs or drug candidates. Many drugs will go through a series of partitioning steps: (a) leaving the aqueous extracellular fluids, (b) passing through lipid membranes, and (c) entering other aqueous environments before reaching the receptor. In this sense, a drug is passing the same partitioning phenomenon that happens to any chemical in a separatory funnel containing water and a non-polar solvent. So a compound must have an optimal lipophilicity, because if the solute is very lipophilic it will remain trapped in the membrane^6^. Lipophilicity is one of the main factors influencing the pharmacokinetic behavior of *β*-blockers by several ways: 1-Oral absorption, 2-Penetration in the central nervous system (CNS), 3-Renal clearance, 4-Degree of biotransformation and plasma half-life, 5-Cardioselectivity, 6-Cornealpenetration^7, 8^. For example, the most lipophilic *β*-blockers (such as propranolol) penetrate readily into the CNS and raise central effects (somnolence), whereas the more hydrophilic drugs have a low CNS penetration and negligible central effects^8^. The *in situ* rat gut technique is an informative tool yielding realistic absorption rates. In 1981 a study of 18 sulfonamides, the absorption rate constant k_a_ was correlated with the lipophilicity parameter^9^. Good gastrointestinal absorption was for many years a problem in the development of Penicillins. Yoshimura^10^ developed an organized study in mice and rats and showed that the two major molecular properties influencing the GI absorption of penicillins are their stability in acidic solutions and their lipophilicity. Corneal penetration is an overcritical condition for the therapeutic success of ocularly administered drugs such as *β*-blockers used as antiglaucoma agents. In 1983, an important study showed that lipophilicity clearly plays a key role in penetration through intact cornea. In a series of 12 *β*-blockers, the logPC (permeability coefficient) exhibited a parabolic relation with lipophilicity^11^. For a homogeneous set of phenols, a parabolic relation was found between human skin permeability (K_p_) and the logP_o/w_^12^. In 1991, for 11 aromatic acids (model compounds and anti-inflammatory drugs) their binding constant to bovine serum albumin (in logarithmic form) was correlated with hydrophobic index obtained by RP-HPLC^13^. In another study, the unbound fraction in plasma (f_u_) that was taken as the biological response, showed a sigmoidal relation with logP_o/w_^14^. Interestingly, parabolic relations between protein binding and lipophilicity are also known, validating the limited dimensions of some binding sites. When large molecules such as Cephalosporins were tested for their association constant (K_a_) to human serum albumin, a fair parabolic relation was found with lipophilicity^15^. In the important study, the concentration of 10 basic drugs in plasma and 8 non-metabolizing tissues was examined administration to rabbits. These drugs were weakly basic benzodiazepines and strongly basic neurological drugs. Good linear relations (R^2^ = 0.92 to 0.97) were found between the tissue-to-plasma concentration ratios of unbound, non-ionized drugs and their logP_o/w_. The slope of the linear regressions raised in the series: muscle < skin < bone < brain < gut < heart < lung < adipose^16^. In many studies on drug permeation through biological membranes (gut wall, skin, blood-brain barrier, and Caco-2 cell monolayer), relationships between permeation and lipophilicity have been developed with homologous series of compounds of a diverse nature (acidic, alkaline and neutral) to investigate the influence of lipophilicity on passive diffusion. For example Sigmoidal relationships were established between permeability coefficients in rat jejunum and logP_o/w_ for seven steroids^17^, and 11 *β*-blockers^18^. Even so, despite the good solubility of most organic compounds in n-octanol and ease in lab handling, the experimental determination of logP_o/w_ remains a resource- and time-consuming process. Methods to estimate logP_o/w_ are basically dedicated to medicinal chemistry and molecular design activities. Estimation approaches involve group and atom contribution methods^19, 20^, quantitative structure property relationships (QSPR) derived from statistical regressions^21–23^. Group and atom contribution models have usually been based on fragments, derived either from atoms or groups of atoms, which are assigned incremental logP_o/w_ contributions^24^. QSPR have been developed as alternate strategies of estimating lipophilicity. The assumption of QSPR for logP_o/w_ is that physicochemical properties can be correlated with molecular structural characteristics (geometric and electronic) expressed in terms of appropriate molecular descriptors^25^. In recent years, enhancements in logP_o/w_ QSPR have been suggested through the use of molecular descriptors derived from semi-empirical Molecular Orbital theory (quantum mechanics) calculations^26^. For example, Bodor^27^, using AM1 semi-empirical MO theory, reported a standard deviation of 0.306 logP_o/w_ for a 18 parameter linear correlation which was developed for estimating lipophilicity for a heterogeneous data set 302 organic compounds. In 1999, Eisfeld and Maurer^28^ proposed a logP_o/w_ correlation with dipole moment, polarizability, electrostatic potential and molar volume as chemical descriptors, based on a heterogeneous set of 202 compounds with a reported standard deviation and maximum absolute error of 0.287, respectively. Yaffe^29^, using Fuzzy ARTMAP and Back-Propagation Neural Networks Based QSPR, Estimated logP_o/w_ for heterogeneous set of 442 organic compounds.

In this work we develop QSPR modeling of logP_o/w_ of 195 substituted aromatic drugs. These drugs are very important in medicinal chemistry, such as: Alprazolam, that is mostly used to treat anxiety disorders, panic disorders, and nausea due to chemotherapy, Dapsone, that is commonly used in combination with Rifampicin and Clofazimine for the treatment of leprosy, Procaine, that is a local anesthetic drug of the amino ester group. It is used primarily to reduce the pain of intramuscular injection of penicillin, and it is also used in dentistry, Warfarin treatment can help prevent formation of future blood clots and help reduce the risk of embolism^30^. In this paper all of 195 drugs are homogeneous set of aromatic drugs.

## Computational approach

All calculations were run on a Dell Inspiron N5010 laptop computer with Intel® Core™ i7 processor with Windows 7 operating system. The molecular structures of all compounds were drawn into the HyperChem 8.0 (Hypercube, Inc., Gainesville, 2011) and pre-optimized using MM^+^ molecular mechanics method (Polak–Ribiere algorithm). The final geometries of the minimum energy conformation were obtained by more precise optimization with the semi-empirical PM3 method, applying a root mean square gradient limit of 0.05 (Kcal.mol-1.Å^−1^), as a stopping criterion for optimized structures. The molecular descriptors were calculated by VLifeMDS (version: 4.4) Software. A GA/MLR algorithm procedure was used for selection of descriptors using QSARINS (QSAINSubria version 2.2.1 2015) software package. MLR was performed by QSARINS.

## Data set selection

For the present study logP_o/w_ of 195 drug compounds was collected from the literature^31^. All molecules exhibited a wide range of lipophilicity (−2.17; 6.03). In order to obtain a validated and, therefore, predictive QSPR model, an available dataset should be divided into the training and test sets. Commonly, this splitting is performed using random and rational splitting methods^32^. The data set was split randomly into 147 training set and 48 prediction set (see Table 1).

**Table 1:** Experimental *log*P_o/w_, Predicted *log*P_o/w_ and Residuals values for train and test set of Aromatic Drugs for MLR model.

Training set				
No	Name	Experimental *log*P_o/w_	Predicted *log*P_o/w_	Residual
1	2-Aminobenzoic acid	1.26	1.1309	0.1291
2	3,5-Dichlorophenol	3.63	3.6918	−0.0618
3	3-Aminobenzoic acid	0.34	0.399	−0.059
4	3-Bromoquinoline	2.91	2.8631	0.0469
5	4-Aminobenzoic acid	0.86	0.5373	0.3227
6	4-Butoxyphenol	2.87	2.7491	0.1209
7	4-Chlorophenol	2.45	2.488	−0.038
8	4-Ethoxyphenol	1.81	1.942	−0.132
9	4-Iodophenol	2.9	2.7765	0.1235
10	4-Methoxyphenol	1.41	1.3653	0.0447
11	4-Pentoxyphenol	3.26	3.1021	0.1579
12	4-Phenylbutylamine	2.39	2.3327	0.0573
13	4-Propoxyphenol	2.31	2.8643	−0.5543
14	5-Phenylvaleric acid	2.92	2.6447	0.2753
15	Acebutolol	2.02	1.8328	0.1872
16	Acetaminophen	0.34	0.6683	−0.3283
17	Acetophenone	1.58	1.4477	0.1323
18	Acetylsalicylic acid	0.9	0.9666	−0.0666
19	Alprazolam	2.61	3.0152	−0.4052
20	Alprenolol	2.99	2.5599	0.4301
21	Aminopyrine	0.85	1.0384	−0.1884
22	Amitriptyline	4.62	4.9183	−0.2983
23	Amlodipine	3.74	3.3935	0.3465
24	Ampicillin	−2.17	−2.0385	−0.1315
25	Atenolol	0.22	0.1532	0.0668
26	Atropine	1.89	1.4201	0.4699
27	Benzoic acid	1.96	2.1432	−0.1832
28	Bifonazole	4.77	4.9596	−0.1896
29	Bisoprolol	2.15	2.0414	0.1086
30	Bromazepam	1.65	2.2939	−0.6439
31	Bumetanide	4.06	4.5235	−0.4635
32	Bupropion	3.21	3.436	−0.226
33	Carazolol	3.73	3.6693	0.0607
34	Carbamazepine	2.45	3.0449	−0.5949
35	Cefadroxil	−0.09	−0.3343	0.2443
36	Cefalexin	0.65	0.5127	0.1373
37	Celiprolol	1.92	2.0377	−0.1177
38	Chlorambucil	3.7	3.2156	0.4844
39	Chloramphenicol	1.14	0.8834	0.2566
40	Chlorothiazide	−0.24	−0.0353	−0.2047
41	Chlorpheniramine	3.39	3.9023	−0.5123
42	Chlorpromazine	5.4	5.4701	−0.0701
43	Chlorprothixene	6.03	5.3408	0.6892
44	Chlorsulfuron	1.79	1.4552	0.3348
45	Chlortalidone	−0.74	−0.1934	−0.5466
46	Ciprofloxacin	−1.08	−1.5556	0.4756
47	Clofibrate	3.65	3.5281	0.1219
48	Clonazepam	3.02	2.8587	0.1613
49	Clonidine	1.57	2.2257	−0.6557
50	Clotrimazole	5.2	5.0106	0.1894
51	Clozapine	4.1	4.0854	0.0146
52	Cocaine	3.01	2.2712	0.7388
53	Codeine	1.19	1.2284	−0.0384
54	Coumarin	1.39	1.3826	0.0074
55	Debrisoquine	0.85	1.1733	−0.3233
56	Desipramine	3.79	4.173	−0.383
57	Diacetylmorphine	1.59	1.6449	−0.0549
58	Diclofenac	4.51	4.7773	−0.2673
59	Diethylstilbestrol	5.07	5.5014	−0.4314
60	Diltiazem	2.89	2.6989	0.1911
61	Diphenhydramine	3.18	3.128	0.052
62	Doxorubicin	0.65	0.8555	−0.2055
63	Enalaprilat	−0.13	1.1457	−1.2757
64	Fenpropimorph	4.93	4.9856	−0.0556
65	Fluconazole	0.5	−0.1396	0.6396
66	Flufenamic acid	5.56	5.1055	0.4545
67	Flumazenil	1.64	1.0018	0.6382
68	Flumequine	1.72	1.7723	−0.0523
69	Furosemide	2.56	2.2861	0.2739
70	Griseofulvin	2.18	2.2831	−0.1031
71	Heptastigmine	4.82	4.6349	0.1851
72	Hydrochlorothiazide	−0.03	−0.309	0.279
73	Hydroflumethiazide	0.54	0.4884	0.0516
74	Hydroxyzine	3.55	3.422	0.128
75	Ibuprofen	4.13	3.75	0.38
76	Imazaquin	1.86	1.4923	0.3677
77	Imipramine	4.39	4.3287	0.0613
78	Indomethacin	3.51	4.3134	−0.8034
79	Ketoconazole	4.34	4.2547	0.0853
80	Labetalol	1.33	2.3242	−0.9942
81	Lidocaine	2.44	2.6036	−0.1636
82	Lormetazepam	2.72	3.1982	−0.4782
83	Mefluidide	2.02	2.0636	−0.0436
84	Meloxicam	3.43	3.411	0.019
85	Melphalan	−0.52	−0.1399	−0.3801
86	Methotrexate	0.54	0.5184	0.0216
87	Methysergide	1.95	2.0114	−0.0614
88	Metipranolol	2.81	2.4265	0.3835
89	Metoclopramide	2.34	1.9124	0.4276
90	Metoprolol	1.95	1.7498	0.2002
91	Nadolol	0.85	1.0663	−0.2163
92	Naproxen	3.24	3.6225	−0.3825
93	Nifedipine	3.17	2.8894	0.2806
94	Niflumic acid	3.88	3.2672	0.6128
95	Nitrendipine	3.59	3.2033	0.3867
96	N-Methylaniline	1.65	1.6284	0.0216
97	Norcodeine	0.69	0.8584	−0.1684
98	Nordiazepam	3.15	2.9419	0.2081
99	Normorphine	−0.17	0.2632	−0.4332
100	Nortriptyline	4.39	4.2362	0.1538
101	Ofloxacin	−0.41	−0.1945	−0.2155
102	Omeprazole	1.8	1.7495	0.0505
103	Oxprenolol	2.51	2.213	0.297
104	Papaverine	2.95	3.6619	−0.7119
105	Penbutolol	4.62	4.3191	0.3009
106	Penicillin V	2.09	1.465	0.625
107	Pentachlorophenol	5.12	4.9701	0.1499
108	Pentamidine	2.08	2.4219	−0.3419
109	Pericyazine	3.65	4.1045	−0.4545
110	Phenazopyridine	3.31	2.9295	0.3805
111	Phenobarbital	1.53	1.5003	0.0297
112	Phenol	1.48	1.2669	0.2131
113	Phe-Phe-Phe	0.02	0.6718	−0.6518
114	Prazosin	2.16	1.8179	0.3421
115	Primaquine	3	3.4409	−0.4409
116	Probenecid	3.7	3.0608	0.6392
117	Procainamide	1.23	1.2642	−0.0342
118	Procaine	2.14	2.1052	0.0348
119	Promethazine	4.05	4.5525	−0.5025
120	Proquazone	3.13	3.8239	−0.6939
121	Quinidine	3.44	3.0699	0.3701
122	Quinine	3.5	2.7869	0.7131
123	Quinmerac	0.78	0.9345	−0.1545
124	Quinoline	2.15	2.062	0.088
125	Rufinamide	0.9	0.4976	0.4024
126	Salicylic acid	2.19	2.0417	0.1483
127	Serotonin	0.53	1.0892	−0.5592
128	Sotalol	−0.47	−0.0212	−0.4488
129	Sulfadiazine	−0.12	−0.1382	0.0182
130	Sulfinpyrazone	2.32	2.537	−0.217
131	Sulindac	3.6	3.038	0.562
132	Tacrine	3.32	2.8079	0.5121
133	Terazosin	2.29	2.332	−0.042
134	Terbutaline	−0.08	0.2173	−0.2973
135	Terfenadine	5.52	5.3235	0.1965
136	Tetracaine	3.51	3.7148	−0.2048
137	Thiabendazole	1.94	1.3245	0.6155
138	Thiamphenicol	−0.27	−0.5873	0.3173
139	Tralkoxydim	4.46	4.4558	0.0042
140	Trazodone	1.66	2.3977	−0.7377
141	Trimethoprim	0.83	1.4642	−0.6342
142	Trovafloxacin	0.15	−0.3398	0.4898
143	Trp-Phe	−0.28	0.2391	−0.5191
144	Trp-Trp	−0.1	−0.018	−0.082
145	Tryptophan	−0.77	−0.2481	−0.5219
146	Verapamil	4.33	3.8853	0.4447
147	Warfarin	3.54	2.4709	1.0691
**Test set**				
148	1-Benzylimidazole	1.6	1.248	0.352
149	2,4-Dichlorophenoxy acetic acid	2.78	2.9783	−0.1983
150	3,4-Dichlorophenol	3.39	3.8638	−0.4738
151	3-Chlorophenol	2.57	2.5277	0.0423
152	Amoxicillin	−1.71	−1.7229	0.0129
153	Antipyrine (phenazone)	0.56	0.4371	0.1229
154	Bentazone	2.83	1.7299	1.1001
155	Benzocaine	1.89	1.9062	−0.0162
156	Carvedilol	4.14	3.4016	0.7384
157	Cromolyn	1.95	1.7931	0.1569
158	Dapsone	0.94	0.9417	−0.0017
159	Diflunisal	4.32	3.9003	0.4197
160	Disopyramide	2.37	2.7188	−0.3488
161	Ephedrine	1.13	0.6715	0.4585
162	Ergonovine	1.67	1.8769	−0.2069
163	Flamprop	3.09	3.117	−0.027
164	Flurbiprofen	3.99	3.9066	0.0834
165	Fluvastatin	4.17	4.302	−0.132

## Computational methods

### Descriptor generation

Molecular descriptors are generated from molecular structures. Although different descriptors utilize different processing steps, still there are numerous steps common to these procedures. Molecular descriptors are powerful tools for the approximation of selected properties of chemical structures in an easy-to-handle form that allows efficient comparison and selection of compounds possessing required chemical, structural, pharmacological or biological features. In this study molecular descriptors were calculated for each compound by the VLifeMDS on the minimal energy conformations. VLifeMDS calculates about 500 different molecular descriptors from the categories: topological, electronic, electrostatic, E-state, information theory based, physicochemical and semi-empirical.

### Descriptor selection

After descriptor generation a pool of the molecules with the corresponding descriptors become available for model calculation. But a limited number of modeling descriptors, related to the studied response, must be selected from the available pool. Descriptor selection is the process of selecting a subset of relevant variables for use in model construction. In QSARINS this is done using a GA/MLR procedure. This technique is able to explore a broad range of solutions, searching for the best ones, by maximizing or minimizing a selected fitness function. This is done mimicking the natural selection, where the best solutions replace the less performing. In biological terms, one would say that the best genes in the population displace the less fitting. In our case, every descriptor represents a gene, and a set of descriptors represents a chromosome. The fitness of a chromosome is related to the matching model performances. Starting with a pool of chromosomes, small subsets of chromosomes are picked randomly, and the best become parents. Couples of parent chromosomes are then crossed at a random position (crossing-over), thus obtaining the offspring, whose chromosomes are a combination of the parent ones. If among the new chromosomes one or more of them outperform the less fitting in the parent population, these chromosomes will replace the less performing. Repeating the aforesaid procedure many times, and introducing also random mutations (descriptor substitution) in the chromosomes, the result at the end of the procedure is a population of models with better performances than the models introduced at the beginning. In order to prevent a completely random beginning of the GA, in QSARINS, the best set of descriptors extracted from the all subset process is used as the core of the chromosomes of the initial population. In QSARINS, the tuning of the GA can be done changing the population size, the mutation rate, and the number of generations. A fundamental option is the selection of the fitness function to be used by GA. In the work, leave-one-out cross-validation (Q^2^_LOO_) was used as fitness function throughout the GA process. When increasing the model size does not improve the Q^2^ value significantly, the GA selection will be stopped. Q^2^_LOO_ used as fitness function, is useable to select models with high fitting with the minimum number of descriptors. However, it is essential to note that they are fitting criteria, so they provide no information on the predictive ability of the models. For this reason, it is here proposed to use Q^2^_LOO_ as fitness function for the selection of predictive models^33^. The important parameters used in the GA process were set as below: population size 100, maximum allowed descriptors in a model 10 and reproduction/mutation trade-off 0.5. Finally, we obtained a 10-descriptor subset, which keeps most interpretive information for logP_o/w_. Four descriptors were calculated for each compound in the data set. The selected descriptors are: SKMostHydrophobic Area, SAHydrophobic Area, SKAverage, XKAverage Hydrophobicity, PSA, Average Potential, Polar Surface Area Excluding P & S, 4Path Count, ChiV6chain and AlphaR.

### Modeling method in QSARINS

The datasets used in QSPR analysis are, as previously mentioned, composed of descriptors that should be correlated with the corresponding experimental responses. At this step it is necessary to apply a quantitative method able to find the existing relationship between a limited number of structural descriptors and the modeled response. In QSARINS, the used method is the MLR approach that can be demonstrated by the following formula:

2 $${{\rm{y}}}_{{\rm{i}}}={{\rm{b}}}_{0}+\sum _{{\rm{j}}=1}^{{\rm{n}}}{{\rm{b}}}_{{\rm{j}}}{{\rm{x}}}_{{\rm{ij}}}+{{\rm{e}}}_{{\rm{i}}}$$

where a linear relationship is computed between the studied responses (y_i_) and the selected values of the descriptors (x_ij_); e_i_ is the random error (called also model residual). The intercept (b_0_) and the coefficients (b_j_) are thus to be evaluated. The equation (2) can be rewritten in a more compact form using the matrix notation:

3 $${\rm{y}}={\rm{Xb}}+{\rm{e}}$$

where y is the responses vector, b the vector of the coefficients and e is the vector of the errors. X is the matrix of the model, where the columns are the descriptors. In this software, to estimate the vector of the coefficients, the OLS technique is used:

4 $$\hat{{\rm{b}}}={({{\rm{X}}}^{{\rm{T}}}{\rm{X}})}^{-1}{{\rm{X}}}^{{\rm{T}}}{\rm{y}}$$

where $\hat{{\rm{b}}}$ is the vector that estimates the b vector of the coefficients, X^T^ the transposed X matrix and ^−1^ is the inverse matrix operation. The OLS minimizes the sum of squares of the difference between the experimental responses and the ones calculated by the model. To work correctly, the OLS assumes that: (1) a linear relationship exists between the descriptors and the response, (2) the response errors are independent and similarly distributed, (3) the descriptors are not too correlated among them, (4) there are more compound than modeling descriptors (a ratio that should be always higher than 5:1). Once the coefficients of the model are calculated, it is possible to obtain the vector of the $\hat{{\rm{y}}}$, as in the following formula:

5 $$\hat{{\rm{y}}}={\rm{X}}\hat{{\rm{b}}}={\rm{X}}{({{\rm{X}}}^{{\rm{T}}}{\rm{X}})}^{-1}{{\rm{X}}}^{{\rm{T}}}{\rm{y}}={\rm{Hy}}\,$$

where H is the leverage (or hat) matrix that relates the calculated and the experimental responses. The diagonal elements of the hat matrix **h**_**ii**_ are useable to determine the distance of the **i** object from the centre of the chemical space of the model^34, 35^, thus, for checking the structural applicability domain (AD) of the model.

### Model evaluation

Evalution of QSPR model is a very important aspect. It is acknowledged that the goodness-of-fit is very important for QSPR models. The quality of goodness-of-fit of the models is quantified by the R^2^ squared correlation coefficient, R^2^_adj_ is adjusted squared correlation coefficient, s is the standard error of the regression and F is the Fisher ratio for regression. R^2^ is a statistic that will give some information about the goodness of fit of a model. R^2^ is defined as:

6 $${R}^{2}=1-\frac{RSS}{TSS}$$

where RSS is the residual sum of squares and TSS is the total sum of squares. Adjusted R^2^ detects the possible overfitting of a model so, used as fitness functions, are useful to select models with high fitting with the minimum number of descriptors. Adjusted R^2^ is defined as:

7 $${R}_{adj}^{2}=1-[(\frac{n-1}{n-m-1})(1-{R}^{2})]$$

where n is the number of members of the training set and m is the number of descriptors included in the model. The Adjusted R^2^ is a better measure of the proportion of variance in the data explained by the correlation than R^2^. The standard error indicates dispersion degree of random error. F-ratio test in regression is defined as the ratio between the variance explained by the model to the residual variance. The larger R^2^, R^2^_adj_ and F, the smaller s, and the model will have more fitting ability.

### Model validation

Model calculation and evaluation are the basic steps in QSPR analysis, but are not sufficient to guarantee the model validity. Validation is fundamental to ensure the reliability of data predicted by the models. Validation of QSPR model is very important aspect, thus internal and external validation is considered to be necessary for model validation^35^.

Internal validation is obtained from analyzing of each one of individual objects that configure the final equation. This procedure is leave-one-out (LOO) cross-validation. This process was done in training set and Q^2^_LOO_ is calculated.

8 $${Q}_{LOO}^{2}\,or\,{Q}_{LMO}^{2}=1-\frac{PRESS}{TSS}$$

where TSS is the total sum of squares that is the sum of squared deviations from the data set mean and PRESS is the sum of squares of the prediction errors. The larger Q^2^_LOO_ and the model will have more predictive ability. However, a perturbation of only one compound at a time is very weak to demonstrate real model robustness. In QSARINS, the stronger Leave-More (or many)-Out (LMO) technique is also included. This technique studies the behavior of the model when a larger number of compounds are eliminated. LMO is used to counteract the slight overoptimism of LOO-cross-validation. The model under analysis can be considered stable if the R^2^ and Q^2^ values calculated in every LMO iteration and their averages (R^2^_LMO_ and Q^2^_LMO_), are close to R^2^_LOO_ and Q^2^_LOO_ values of the model^36^.

To show that the model is not the result of chance correlation, the Y-scrambling procedure can be applied. In this process, the responses are shuffled at random, so no correlation between them and the descriptors should exist. As a consequence, the performances of the corresponding scrambled models should decrease drastically. In this case if the original model under validation is good, the values of R^2^ and Q^2^ of the every iteration, and their averages (R^2^_yscr_ and Q^2^_LOO-yscr_), must be far and much smaller from the values of the original model. If Q^2^_LOO-yscr_ < 0.2, and R^2^_yscr_ < 0.2, there is no risk of chance correlation in the developed model.

In the process of model validation, external validation is necessary. External validation of the model is checked for its ability to predict new compounds. This is done by applying the model equation, obtained on the training set, to one or more prediction data set(s), that is the excluded compounds that have never been used in model calculation, and measuring the performances by means of different criteria, such as: RMSE^37^, Q^2^_F1_^38^, Q^2^_F2_^39^, Q^2^_F3_^40^, CCC^41^ and Q^2^_EXT_^42^.

The external Q^2^_F1_ for the test set is determined with the following equation:

9 $${Q}_{F1}^{2}=1-\frac{PRESS}{S{S}_{EXT}({\bar{y}}_{TR})}$$

where ${\bar{y}}_{TR}$ indicates the response means of the training set, respectively. PRESS is the predictive sum of squares, $S{S}_{EXT}({\bar{y}}_{TR})\,\,$ is the total sum of squares of the external set calculated by means of the training set mean, respectively. Consequently, this formula gives valid values when the test set spans the whole response domain of the model because in this case the test set mean approaches the training set mean.

Q^2^_F2_ is defined as:

10 $${Q}_{F2}^{2}=1-\frac{PRESS}{S{S}_{EXT}({\bar{y}}_{EXT})}$$

where ${\bar{y}}_{EXT}$ indicates the response means of the external test set and $S{S}_{EXT}({\bar{y}}_{EXT})$ is the total sum of squares of the external set calculated by means of the external set mean, respectively. Function Q^2^_F2_ does not account for information about the reference model because ${\bar{y}}_{EXT}$ encodesinformation derived from the external set and this informationalters continuously on the basis of the objects belonging to the external set.

Q^2^_F3_ is defined as:

11 $${Q}_{F3}^{2}=1-\frac{PRESS/{n}_{EXT}}{TSS/{n}_{TR}}$$

where TSS is the total sum of squares n_EXT_ is number of test set and n_TR_ is number of train set. Expression Q^2^_F3_ reduces to expression for Q^2^_LOO_ when training and test sets coincide (n_EXT_ = n_TR_), or, in other words, when all available data are used both for fitting and assessing model predictive ability.

CCC: Concordance correlation coefficient.

12 $$CCC=\frac{2\,{\sum }_{i=1}^{n}({x}_{i}-\bar{x})({y}_{i}-\bar{y})}{{\sum }_{i=1}^{n}{({x}_{i}-\bar{x})}^{2}+{\sum }_{i=1}^{n}{({y}_{i}-\bar{y})}^{2}+n{(\bar{x}-\bar{y})}^{2}}$$

It is well suited to measure the consensus between experimental and predicted data, which should be the real aim of any predictive QSPR models. Where x_i_ and y_i_ correspond to the abscissa and ordinate values of the graph plotting the prediction experimental data values vs. the ones calculated using the model. Where n is the number of chemicals, and $\bar{x}$ and $\bar{y}$ correspond to the averages ofabscissa and ordinate values, respectively. This coefficient measures both precision (how far the observations are from the fitting line) and accuracy (how far the regression line deviates from the slope 1 line passing through the origin, the concordance line), consequently any divergence of the regression line from the concordance line gives as a consequence a value of CCC smaller than 1.

An elemental property of a function for the assessment of model fit from external evaluation data is that external observations are independent of each other. This means that the Q^2^ value derived from the whole external data set Q^2^_EXT_ and the average of the Q^2^ values obtained taking separately each external data one at one time should coincide. The optimized model was applied for the prediction of logP_o/w_ values of 49 drugs in the prediction set which were not used in the optimization procedure. The predictive ability of a model on external validation set can be expressed by Q^2^_EXT_.

13 $${Q}_{EXT}^{2}=\frac{{\sum }_{i=1}^{{n}_{EXT}}{Q}_{i}^{2}}{{n}_{EXT}}$$

where Q^2^_i_ is the external Q^2^ calculated taking into account only the ith object of the test set and n_EXT_ is the total number of external objects.

An additional measure of the accuracy of the proposed QSPR is the RMSE (root mean squared errors) that summarizes the overall error of the model.

14 $$RMSE=\sqrt{\frac{{\sum }_{i=1}^{{n}_{EXT}}{({\bar{y}}_{i}-{y}_{i})}^{2}}{{n}_{EXT}}}$$

where ${\bar{y}}_{i}$ is the predicted value for the ith test object and y_i_ its observed value, n_EXT_ is the total number of test objects. This parameter depends only on the mean deviations between predictions and observed values and it can always be calculated even when there is only one test object. It is calculated as the square root of the sum of squared errors in prediction divided by their total number. This parameter was calculated to compare the accuracy and the stability of our models in the training (RMSE_TR_) and in the prediction (RMSE_EXT_) sets. It is important to note that RMSE values must not only below but also as similar as possible for the training, cross-validation and external prediction sets. This suggests that the proposed model has both predictive ability (low values) as well as sufficient generalizability (similar values).

The AD is a theoretical area in chemical space, defined by the model descriptors and modeled response, and thus by the nature of the chemicals in the training set, as represented in each model by specific molecular descriptors As even a robust, significant and validated QSPR cannot be expected to reliably predict the modeled property for the all universe of chemicals, its domain of application must be defined, and the predictions for only those chemicals that fall in this domain can be considered reliable. The Williams plot of the regression permits a graphical detection of both the outliers for the response and the structurally influential chemicals in a model. The Williams plot detects the outliers for the response (Y-outliers) and those for the structure (X-outliers). It consists of plotting the standardized residuals on the y-axis and the leverage values from the hat matrix diagonal on the x-axis. The leverage (h) of a compound measures its influence on the model. The leverage of a compound in the original variable space is defined as:

15 $${\rm{H}}={\rm{X}}{({{\rm{X}}}^{{\rm{T}}}{\rm{X}})}^{-1}{{\rm{X}}}^{{\rm{T}}}$$

where the X is the model matrix derived from the training set descriptor values and the leverage values of training set are diagonal elements of the Hat or Influence matrix H **(**h_i_ = diag(H)). The leverage values are always between 0 and 1. The warning leverage *h* ^*^ is defined as follows:

16 $${h}^{\ast }=3\times \frac{{\sum }_{i}{h}_{i}}{n}=3\times \frac{p^{\prime} }{n}\,(i=1,\ldots ,\,n)$$

where n is the number of training set compounds and *p*′ is the number of model parameters plus one. Observations with standardized residuals greater than (−3; +3) range, which lie outside the horizontal reference lines on the plot, are outlier’s responses in the QSARINS (standardized residuals >$\pm 3\sigma$ is the standard deviation of residuals). Standardized residual (SR_i_) for each sample is calculated as in equation (17):

17 $$S{R}_{i}=\frac{({y}_{i}-{\hat{y}}_{i})}{\sqrt{\frac{{\sum }_{i=1}^{n}{({y}_{i}-{\hat{y}}_{i})}^{2}}{n}}}$$

where y_i_ and ${\hat{y}}_{i}$ are respectively the measured and predicted values of the property; n is the number of compounds in each set of data. To visualize the AD of a QSPR model, the plot of standardized residuals versus leverage values (*h*) (Williams plot) can be used for an immediate and simple graphical detection of both the response outliers and structurally influential chemicals in a model (*h* > *h* ^*^). Concerning the residuals, all the chemicals falling above or below the user defined threshold are not well predicted and thus considered as outliers. Too many outliers, especially those underestimated, are symptomatic of a poor model and this is the reason of implementing the counting of the outliers. Leverage values represent the degree of influence that the structure of every single chemical has on the model. A compound with high leverage in a QSPR model is the driving force for the variable selection if this compound is in the training set (good leverage). A high leverage compound in the prediction set is detected as far from the chemical domain of the training compounds, thus it could lead to unreliable predicted data, being the result of substantial extrapolation of the model. Therefore, the structural information of the chemicals included in the training set could be not sufficient for a reliable prediction of chemicals lying outside of the training-AD^43^.

## Results and Discussions

### Multiple regression analysis

The MLR analysis was used to derive a QSPR model. The data set was randomly divided into training and test set. 147 drugs were selected as the training set in the modeling. 48 drugs were chosen as a prediction set and were used for external validation of the MLR. Making use of the MLR method, the linear model was obtained, in which the molecular descriptors were used as independent variables. In the Table 2, the list of descriptors, their coefficients and model parameters have been shown.

**Table 2:** The list of descriptors, their coefficients and model parameters.

No.	Descriptor	Coefficient	Model parameter
1	Intercept	−2.1502	n = 147
2	PSA	−0.0176	R^2^ = 0.9433
3	SKMostHphobic	7.1814	R^2^_adj_ = 09391
4	4PathCount	−0.0108	s = 0.4031
5	chiV6chain	6.4751	F = 226.3247
6	Average Potential	−15.9893	
7	AlphaR	−0.0897	
8	XKAverageHydrophobicity	2.1153	
9	SAHydrophobic Area	0.0055	
10	SKAverage	−4.0213	
11	Polar Surface Area Excluding P&S	0.0176	

**Figure 1:**
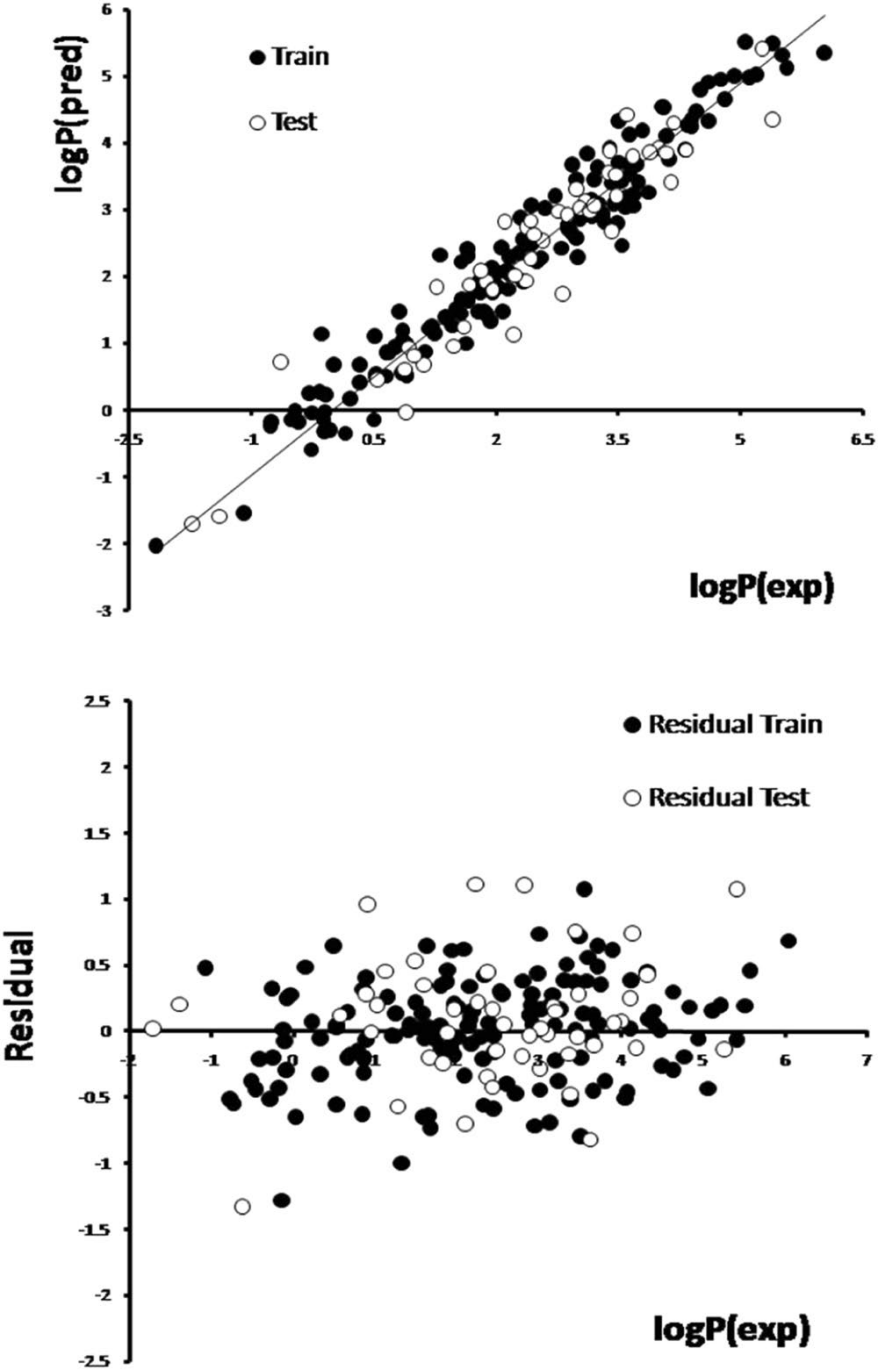
(**A)** Plot of predicted versus experimental of logP_o/w_ values. (**B**) Plot of residual versus experimental of logP_o/w_ values.

**Figure 2:**
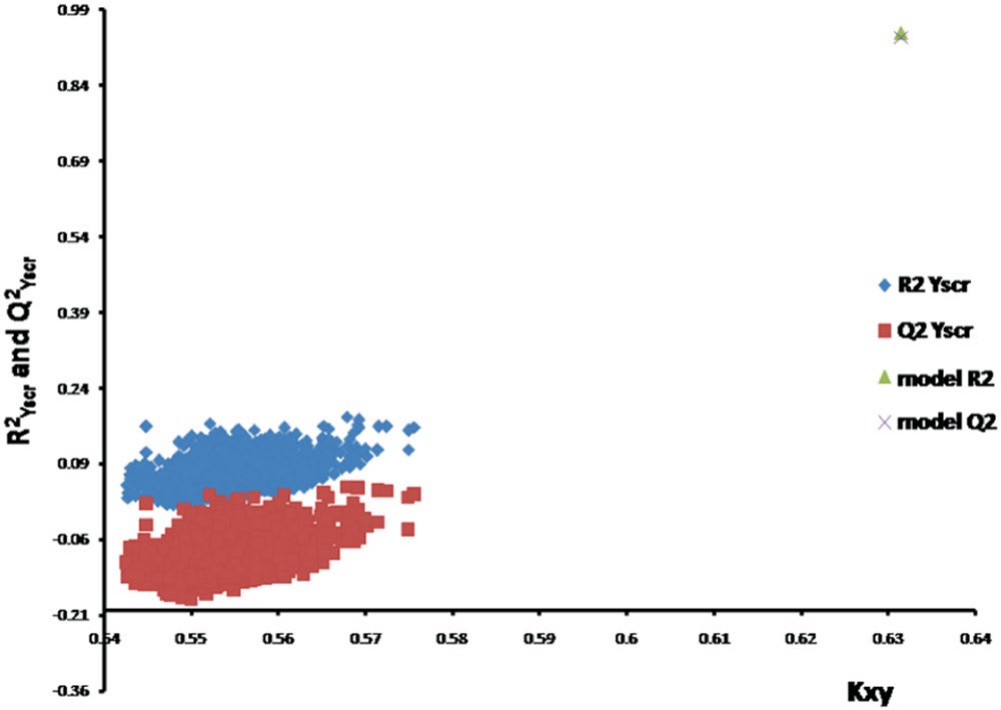
Plot of R^2^ and Q^2^ Y-scrambling models versus correlations among the block of the descriptors and the experimental data (Kxy).

**Figure3:**
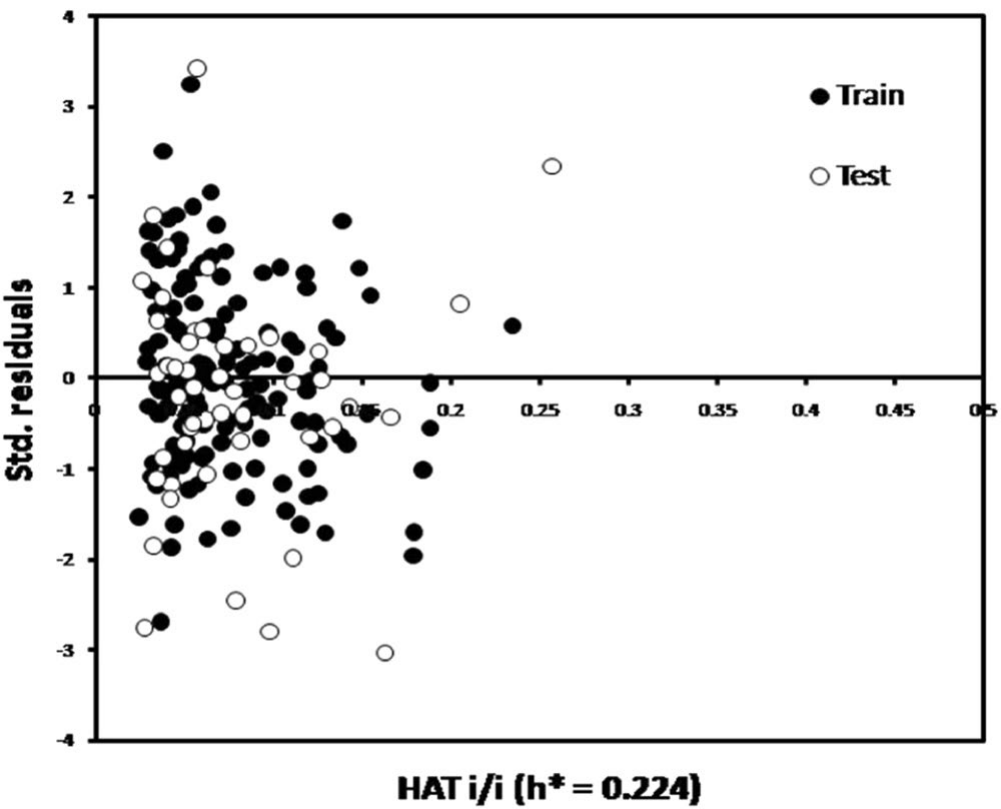
William plot of standardized residual (SR) versus leverage (h) values for training and test sets.

Where, n is the number of compounds used for regression, R^2^ is the squared correlation coefficient, R^2^_adj_ is adjusted squared correlation coefficient, s is the standard error of the regression and F is the Fisher ratio for regression. R^2^ is a measure of how well the regression line approximates the real data points. The high R^2^ (R^2^ = 0.9433) indicates that the regression line perfectly fits the data. The squared correlation coefficient values closer to 1 represents the better fit of the model. Equation 18 has R^2^_adj_ value of 0.9391, which indicates very good agreement between the correlation and the variation in the data. s represents the average distance that the observed values fall from the regression line. Conveniently, it tells you how wrong the regression model is on average using the units of the response variable. Smaller values (s = 0.4031) are better because it indicates that the observations are closer to the fitted line. High values of the F (F = 226.3247) indicate that the model is statistically significant. The F-test reflects the ratio of the variance explained by the model and the variance due to the error in the model, and high values of the F-test indicate the model is statistically significant. The predicted and experimental values of logP_o/w_, residuals (experimental logP_o/w_ − predicted logP_o/w_), are presented in Table 1. The plots of predicted logP_o/w_ versus experimental logP_o/w_, the residuals versus experimental logP_o/w_ value obtained by the MLR modeling and the random distribution of residuals about zero mean are shown in Fig. 1A and B. These results show that the predicted values are in good agreement with the experimental values. The leave-one-out and leave-many-out cross validations were performed in training set. The Q^2^_LOO_ and Q^2^_LMO_ describe the stability of a regression model obtained by focusing on sensitivity of the model to the elimination of any or more data point. (Q^2^_LOO_ = 0.9341, Q^2^_LMO_ = 0.9318 illustrate the stability of the model). In the present study, R^2^_yscr_ = 0.0685 and Q^2^_LOO-yscr_ = −0.0901 show that the model is not the result of chance correlation (see Fig. 2). The external validation is an indispensable validation method used to determine the true predictive ability of the QSPR model. The large value of Q^2^_EXT_ = 0.8982, Q^2^_F1_ = 0.8941, Q^2^_F2_ = 0.8921, Q^2^_F3_ = 0.9118 and CCC = 0.9463 illustrate the predictive capability of a model on external prediction set. In the Williams plot for AD (see Fig. 3), Sulfasalazine in the test set is to the right of the vertical line, which indicates it has high leverage value (*h* > *h* ^*^ = 0.224) and low standardized residual, it is belong to the model AD. The chemical compound of Doxorubicin in the training set is to the right of the vertical line, which indicate they have high leverage value (*h* > *h* ^*^ = 0.224) and low standard residual. These chemicals with high leverages have a stronger influence on the model than other chemicals, and they are influential. In the standardized residuals plot, Enalapilat in training set and Phe-Phe in test set have standard residual > (−3; +3) range, which confirms that there are two outliers. Furthermore, there is no clear pattern in the residuals, so nothing seems to be wrong with the model. The fitting criteria, internal validation criteria and external validation criteria are shown in Table 3.

**Table 3:** Fitting, internal validation and external validation criteria for GA/MLR model.

Criteria	Statistical parameters		
Fitting criteria	R^2^: 0.9433	RMSE_TR_: 0.3877	${\rm{S}}$: 0.4031
	${{\rm{R}}}_{{\rm{adj}}}^{2}$: 0.9391		${\rm{F}}$: 226.3247
Internal validation criteria	${{\rm{Q}}}_{{\rm{LOO}}}^{2}$: 0.9341	RMSE_CV_: 0.4181	${{\rm{R}}}_{{\rm{yscr}}}^{2}$: 0.0685
	${{\rm{Q}}}_{{\rm{LMO}}}^{2}$: 0.9318		${{\rm{Q}}}_{{\rm{yscr}}}^{2}$: −0.0901
External validation criteria	${{\rm{Q}}}_{{\rm{EXT}}}^{2}$: 0.8982	RMSE_EXT_: 0.4836	
	${{\rm{Q}}}_{{\rm{F}}1}^{2}$: 0.8941		
	${{\rm{Q}}}_{{\rm{F}}2}^{2}$: 0.8921		
	${{\rm{Q}}}_{{\rm{F}}3}^{2}$: 0.9118		
	CCC_EXT_: 0.9463		

## Interpretation of descriptors

### SKMostHydrophobic Area, SAHydrophobic Area and SKAverage

SKMostHydrophobic Area is the most hydrophobic value on the van der Waals (vdw) surface. The van der Waals surface of a molecule is a surface might reside for the molecule based on the hard cutoffs of van der Waals radii for individual atoms, and it represents a surface through which the molecule might be conceived as interacting with other molecules. Hydrophobicity (also termed hydrophobic) materials possessing this characteristic have the opposite response to water interaction. Compared to hydrophilic materials, hydrophobic materials (water hating) have little or no tendency to absorb water and water tends to bead on their surfaces. Hydrophobic materials possess low surface tension values and lack active groups in their surface chemistry for formation of hydrogen-bonds with water. Hydrophobicity is very important in solubility of drugs. Accordingly drugs that are extremely hydrophobic are also poorly absorbed, because they are totally insoluble in aqueous body fluids and, therefore, cannot gain access to the surface of cells. For a drug to be readily absorbed, it must be largely hydrophobic, yet have some solubility in aqueous solutions. This is one reason why many drugs are weak acids or weak bases. There are some drugs that are highly lipid-soluble, and they are transported in the aqueous solutions of the body on carrier proteins such as albumin. The results indicate that the SKMostHydrophobic Area increases as logP_o/w_ increases. SAHydrophobic Area is van der Waals surface descriptor showing hydrophobic surface area. Lipid solubility of a compound is of special importance to drug discovery and development, because it is directly related to the transport abilities of a drug candidate to cross biological membranes. The requirement is that drug molecules must be soluble enough in lipid to get into membranes but cannot be so soluble that they become trapped in the membranes. These membranes are not exclusively anhydrous fatty or oily structures. As a first approximation, membranes can be considered bi-layers composed of lipids consisting of a polar cap and large hydrophobic tail. Phosphoglycerides are major components of lipid bi-layers. Other groups of bi-functional lipids include the sphingomyelins, galactocerebrosides, and plasmalogens. The hydrophobic portion is composed largely of unsaturated fatty acids, mostly with cis double bonds. In addition, there are considerable amounts of cholesterol esters, protein, and charged mucopolysaccharides in the lipid membranes. The final result is that these membranes are highly organized structures composed of channels for transport of important molecules such as metabolites, chemical regulators (hormones), amino acids, glucose, and fatty acids into the cell and removal of waste products and biochemically produced products out of the cell. Apparently, increasing the SAHydrophobic Area increases logPo/w. SKAverage is the Average hydophobicity function value. According to Supplementary information, some molecules have a positive Hydrophobicity function, others are negative. If the desired compound is more soluble in non-polar than polar phase, the Average hydophobicity function value is higher. Finally, increasing the SKAverage increases logP_o/w_. SKMostHydrophobic Area, SAHydrophobic Area and SKAverage are calculated by SlogP method^44^. This method represents a new atom type classification system for use in atom-based calculation logP_o/w_.

### XKAverageHydrophobicity

XKAverageHydrophobicity is the Average hydrophobic value on the van der Waals (vdw) surface. This descriptor is calculated by XlogP method^45^. In this method the atoms are classified by their hybridization states and their neighboring atoms. XlogP is based on the summation of atomic contributions and includes correction factors for some intra-molecular interactions. The XKAverageHydrophobicity increases as logP_o/w_ increases.

### PSA, Polar Surface Area Excluding P & S and Average Potential

Polar surface area of a molecule is defined as the sum of the contributions to the molecular surface area of polar atoms such as oxygen, nitrogen and their attached hydrogen’s. This parameter is easy to understand and, most importantly, provides good correlation with experimental transport data. PSA is a descriptor showing the correlation with passive molecular transport through membranes, which allows prediction of human intestinal absorption, caco-2 mono-layer permeability, and blood-brain barrier penetration. Molecules with a polar surface area of greater than 140 angstrom squared tend to be poor at permeating cell membranes. For molecules to penetrate the blood-brain barrier a PSA less than 90 angstroms squared is usually needed. In new approach, PSA is calculated based on the summation of tabulated surface contributions of polar fragments by Ertl^46^. PSA increases as logP_o/w_ decreases. Polar Surface Area Excluding P & S signifies total polar surface area excluding phosphorous and sulphur. According to Table 2, this descriptor has a positive coefficient. This shows that the molecules have S and P, tend to dissolve in polar phase. In contrast, the molecules that have other atoms tend to dissolve in non-polar phase. Thus, the presence of S and P atoms in the molecules are not in favor of the lipophilicity. Polar Surface Area Excluding P & S increases as logP_o/w_ increases. Average Potential signifies average of the total electrostatic potential on van der Waals surface area of the molecule. According to Table 2, Average Potential increases as logP_o/w_ decreases.

### 4PathCount, ChiV6chain and AlphaR

4Path count signifies total number of fragments of fourth order (four bond path) in a compound. This descriptor signifies total number of fragments of fourth order (four bond path) in a compound. 4Path Count describes the connectivity of the atoms within the molecule and also explains its branching and flexibility or rigidity. In fact, lipophilicity decreases with branching. This is due to the fact that the branching of the chain makes the molecular most compact and thereby decreases the surface area. Thus, more branching will reduce the size of the molecule, making it harder to solvate in non-polar phase. As a result, the lipophilicity of the normal compound isomers is higher in all instances than the branched compounds. According to Table 2, 4Path Count shows a negative coefficient towards the lipophilicity, which indicates this descriptor increases as logP_o/w_ decreases. ChiV6chain signifies atomic valence connectivity index for six membered rings. This descriptor indicates the importance of molecular bulk for lipophilicity. Lipophilicity increases with molecular bulk because large molecules are better solved in non-polar phase such as n-octanol. This descriptor is calculated by molecular graph. Apparently, increasing the chiV6chain increases logP_o/w_. AlphaR indicates sum of *α* value of all non-hydrogen atoms in a reference alkane. The reference alkane is when all heteroatoms in the molecular graph are replaced by carbon and multiple bonds are replaced by single bonds, corresponding molecular graph may be considered as the reference alkane. The parameter *α* is related to the size of an atom. The term ∑*α* is a measure of molecular bulk. When ∑*α* is compared to that of the corresponding reference alkane, a measure of the heteroatom count and size of a molecule can be obtained.

18 $$\alpha =\frac{Z-{Z}^{v}}{{Z}^{v}}.\frac{1}{PN-1}$$

Where, Z and Z^v^ represent atomic number and valence electron number respectively. The *PN* stands for period number. Hydrogen atom is considered as reference, *α* for hydrogen is taken to be zero. Table 4 shows that *α* value of different atoms. According to Table 2, the coefficient of AlphaR is negative. These results indicate the electronegativy of atoms must be considered. If the molecules that have the atoms such as Cl, Br, S and P, have the higher *α* and increases size and electronegativy. As a result, more electronegative molecules are solved in the aqueous phase^47^. Finally AlphaR increases as logP_o/w_ decreases.

**Table 4:** The list of *α* of atoms commonly occurring in organic compound.

No	atom	*α*
1	H	0.000
2	C	0.500
3	N	0.400
4	O	0.333
5	F	0.286
6	P	1.000
7	S	0.833
8	Cl	0.714
9	Br	1.333
10	I	1.643

## Conclusion

In this work, the MLR was used to construct linear QSPR model to predict logP_o/w_ of a wide and homogeneous set of aromatic drugs. MLR method could model the relationship between logP_o/w_ and descriptors. The GA/MLR method is applied for descriptor selection. The results show that the GA/MLR method is a very effective descriptor selection approach for QSPR analysis. The results indicate that the goodness of fit, robustness and predictive ability of MLR model was perfect from internal and external validation. By performing model validation, it can be concluded that the presented model is valid model and can be effectively used to predict the logP_o/w_. Moreover, the mechanism of the model was interpreted and the applicability domain of the model was defined.

## Electronic supplementary material

Data set1
